# Lone parents, health, wellbeing and welfare to work: a systematic review of qualitative studies

**DOI:** 10.1186/s12889-016-2880-9

**Published:** 2016-02-25

**Authors:** Mhairi Campbell, Hilary Thomson, Candida Fenton, Marcia Gibson

**Affiliations:** MRC/CSO Social and Public Health Sciences Unit, University of Glasgow, 200 Renfield Street, Glasgow, G2 3QB UK

**Keywords:** Lone parent, Welfare to work, Health, Wellbeing, Systematic review, Qualitative synthesis, Welfare reform

## Abstract

**Background:**

Lone parents and their children experience higher than average levels of adverse health and social outcomes, much of which are explained by high rates of poverty. Many high income countries have attempted to address high poverty rates by introducing employment requirements for lone parents in receipt of welfare benefits. However, there is evidence that employment may not reduce poverty or improve the health of lone parents and their children.

**Methods:**

We conducted a systematic review of qualitative studies reporting lone parents’ accounts of participation in welfare to work (WtW), to identify explanations and possible mechanisms for the impacts of WtW on health and wellbeing. Twenty one bibliographic databases were searched. Two reviewers independently screened references and assessed study quality. Studies from any high income country that met the criteria of focussing on lone parents, mandatory WtW interventions, and health or wellbeing were included. Thematic synthesis was used to investigate analytic themes between studies.

**Results:**

Screening of the 4703 identified papers and quality assessment resulted in the inclusion of 16 qualitative studies of WtW in five high income countries, USA, Canada, UK, Australia, and New Zealand, covering a variety of welfare regimes. Our synthesis found that WtW requirements often conflicted with child care responsibilities. Available employment was often poorly paid and precarious. Adverse health impacts, such as increased stress, fatigue, and depression were commonly reported, though employment and appropriate training was linked to increased self-worth for some. WtW appeared to influence health through the pathways of conflict and control, analytical themes which emerged during synthesis. WtW reduced control over the nature of employment and care of children. Access to social support allowed some lone parents to manage the conflict associated with employment, and to increase control over their circumstances, with potentially beneficial health impacts.

**Conclusion:**

WtW can result in increased conflict and reduced control, which may lead to negative impacts on mental health. Availability of social support may mediate the negative health impacts of WtW.

**Electronic supplementary material:**

The online version of this article (doi:10.1186/s12889-016-2880-9) contains supplementary material, which is available to authorized users.

## Background

Lone parents and their children have poor health and social outcomes, disproportionately experiencing depression [1, 2], psychiatric disease, attempted suicide, alcohol and drugs-related disease [3], poor educational outcomes [4], and school behaviour problems [5]. Lone mothers in the UK are twice as likely as partnered mothers to describe their health as ‘not good’ (13 % compared to 7 %) [5]. Much of these adverse outcomes can be attributed to high rates of poverty among lone parents [6–9]. In 2014, 42 % of children in UK lone parent households were poor compared to 23 % in couple households [10]. In many high income countries, employment rates are lower for lone parents than couple parents [9]. In the UK, 63 % of lone parents were in employment compared to 72 % of partnered mothers in 2014 [11]. Governments around the world have attempted to address the issue of lone parent poverty by implementing policies designed to promote employment.

Government policies to promote employment include making receipt of welfare benefits conditional upon efforts to find work. Beginning in the United States in the 1990s, eligibility restrictions, often based on the age of the youngest child, have been introduced in many other Organisation for Economic Cooperation and Development (OECD) countries [12]. The age of youngest child when lone parents are expected to seek employment varies between countries, from under one year old in some US states, and is currently 5 years in the UK [12]. Such welfare to work interventions (WtW) require benefit claimants to prove that they are actively seeking employment, or to participate in training programmes intended to improve employability. Failure to comply with these requirements can lead to financial sanctions. In addition to poverty reduction, rationales for these policies include reducing public expenditure [13] and improving health [14]. Employment may promote increased income, improved parental confidence and consequently enhanced parenting [15]. However, available evidence suggests that employment does not necessarily reduce poverty among lone parents [16]. Despite high employment among lone mothers in Sweden, lone mothers have worse self-reported health than partnered mothers [17], and participating in welfare to work in the USA has been found to reduce cases of anxiety but increase those of depression, with variance among subpopulations [18]. A substantial body of experimental studies on the impacts of WtW on lone parents and their children is currently being synthesised in a systematic review [19]. Twelve randomised controlled trials are included in the review and a preliminary synthesis indicates that impacts on economic outcomes and on measures of adult and child health are small but mostly positive [20].

The impacts of participation in WtW on the health and wellbeing of lone parents and their children, and the mechanisms involved, are unclear. This study contributes to understanding of these by systematically reviewing qualitative studies reporting lone parents’ accounts of participating in WtW, focussing on identifying mechanisms linking their participation with health, wellbeing, and socio-economic determinants of health. Evidence from qualitative studies can provide insights into the mechanisms linking interventions with health and wellbeing [21], and into respondents’ experiences of the intervention. It can also further understanding of the influence of contexts and personal characteristics on individual responses to the intervention [22].

## Methods

For this systematic review, the inclusion criteria were studies that included lone parents; who were participating in WtW programmes; and reported data on health or wellbeing. The review included studies of lone parents and their dependent children living in OECD countries with established social welfare systems. As definitions of lone parents and dependent children can vary slightly between countries and interventions, the review included studies involving lone parents and their children as defined by the study authors. Studies were excluded where there was a mix of lone and couple parents or where the co-habitation status of the parent was unclear. Mandatory WtW interventions were included; studies where participation in the WtW initiative was voluntary and there was no link with benefit eligibility were excluded. Studies were included if there was reference to health or wellbeing (as defined by the study author). Particular areas of interest were experiences and accounts of WtW interventions in relation to the health and wellbeing of participants and their children, and to social determinants of health. Health and wellbeing were conceptualised broadly to encompass stress levels, energy, impact on relationships, managing everyday tasks, and confidence, in addition to physical or mental health conditions. Research from any discipline or theoretical tradition that used recognised qualitative methods of data collection and analysis was included. In accordance with good practice for systematic reviews, the study protocol is available [23], and PRISMA reporting guidelines were used [24].

### Literature search and screening

The search strategy was developed by CF, an information scientist, with contribution from MG. CF conducted the searches in 2009 and 2013. Key search terms were selected to source literature on ‘lone parents’ and ‘welfare to work’. Additional file 1 provides an example search strategy, search terms, and a full list of databases searched. A full search strategy for each electronic database is available from the authors. Twenty one electronic bibliographic databases of peer reviewed articles and grey literature were searched with no date or language limits. For non-English language texts, we were able to ascertain either by the title or English language abstract whether articles were relevant to the review. Two reviewers (MG and MC) independently screened the search results by title, then by abstract. The full text was then screened to establish inclusion decisions. Disagreements were resolved by discussion within the review team.

### Quality assessment of qualitative studies

The quality assessment criteria for qualitative studies were based on those developed by Dixon-Woods et al. [25]. The criteria focus on the transparency and appropriateness of methods used (see Additional file 2). Each study was assessed independently by MG and MC to ascertain whether the research questions, sampling, data collection and analysis were clearly reported and suited to qualitative enquiry, claims made were supported by sufficient evidence, and the paper made a useful contribution to the review question. The results were compared and any differences re-examined and resolved through discussion. Studies were excluded if they did not report any qualitative data, did not use qualitative methods for analysis, or did not make a useful contribution to the review question as assessed by the reviewers.

### Extraction and synthesis

The full text of included studies was imported into NVivo software. Analysis of the extracted data drew on thematic synthesis, a methodology designed to enhance the transparency of synthesising qualitative data and facilitate the construction of new analytical themes from the collated data [26]. Each reviewer (MC, MG, HT) independently assessed three included papers then discussed initial thoughts on broad descriptive coding themes. Line-by-line coding by MC on the findings and discussion sections of six papers identified 30 codes. These codes were organised into five broad descriptive themes, based on the content of the codes and the authors’ knowledge of socio-economic determinants of health. These were then used by MC to conduct line-by-line coding of the remaining included papers. The reviewers met regularly to discuss and agree coding as it developed. A summary of the coded text was collated by MC. This summary was then used by MC, MG and HT to identify analytical themes emerging from the descriptive themes across the included studies, in accordance with the interpretive stage of thematic analysis [26].

## Results

**Fig. 1:**
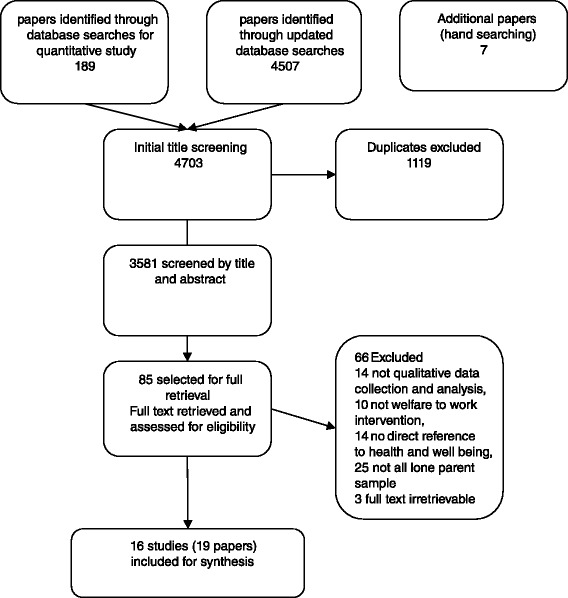
Flow chart of literature search

The searches identified 4703 papers. Following screening, we identified 19 articles reporting 16 studies of compulsory WtW interventions or programmes (Fig. 1), which met the inclusion criteria for the review. Seven were conducted in the USA, three in Canada, three in the UK, two in Australia and one in New Zealand, totalling 724 participants. While the studies met the quality criteria, the recruitment processes of several were ambiguous, and there was variation in the depth of useful information. The results of the quality assessment are shown in Online Appendix B. Studies focused on a variety of aspects of the experience of lone parents involved in WtW. While not all studies reported on every aspect of interest to the review, all presented data for the synthesis. Table 1 provides an overview of the research questions, focus, and methods of the included studies. Many participants moved frequently between WtW and employment. Therefore, respondents and study authors often did not distinguish between the impacts of participating in WtW and being in employment. Further, for many WtW participants the demands of WtW and employment were similar, again leading to a lack of differentiation between the two scenarios. Nonetheless, we aimed to maintain clarity on whether any impacts described were attributed to WtW or subsequent employment.

**Table 1:** Included studies of lone parents’ experience of compulsory welfare to work

Study papers	Country	Year	Data collection	Recruitment	Sample no.	Focus of paper(s)
Baker 2002 [44], 2004; Baker & Tippin 2002 [31]	New Zealand	2001	Face to face interviews	All eligible claimants in study area invited	120	2002, 2004: impact of poor health on gaining and maintaining employment
						2002: demands of meeting parenting, welfare and work requirements
Breitkreuz et al. 2010 [27]	Canada	2001, 2002	Face to face interviews	Via social service agencies, employability programmes and snowballing	17	Impact of unpaid domestic duties and employment for welfare to work lone parents
Critelli et al. 2010 [38]	USA	Prior to 2007	Telephone interviews	Eligible claimants on foster agency lists	100	Impact of welfare to work policies on lone foster parents
Good Gingrich 2010 [39]	Canada	2006 - 2010	Face to face interviews (peer)	Purposive sampling	42	“lone mothers’ experiences of the design, delivery, and enforcement of workfare”
Grahame & Marston 2012 [40]	Australia	2008, 2009	Interviews	Purposive sampling from eligible participants of welfare to work records	21	Wellbeing of welfare to work lone parents: dependency and development of autonomy
Haux et al. 2012 [37]	UK	2009, 2010	Face to face interviews (peer)	Single Parent Action Network participants, Citizens Advice and Job Centre Plus invite, social network sites	50	Experience of welfare to work assistance and implications for wellbeing
Hildebrandt 2002 [34]; Hildebrandt & Kelber 2005 [28]	USA	1999 - 2000	Face to face interviews (peer)	Snowball sampling	34	2002: Effect of welfare to work on lone parents’ health and wellbeing
						2005: Perceptions of lone parents of their health and wellbeing while on welfare to work
Hildebrandt 2006 [29]	USA	2000	Face to face interview	Purposive sampling from participants in work-based welfare programme, snowballing	31	Barriers to maintaining welfare to work participation
Hildebrandt & Ford 2009 [32]	USA	2007 - 2009	Face to face interviews	Community based purposive sampling	41	Barriers to success when lone parents are removed from welfare after the 5 year time limit
Lane et al. 2011 [33]	UK	2011	Interviews	Welfare to work records	60	Experience of welfare to work
McArthur et al. 2013 [41]	Australia	2009	Telephone interviews, focus groups	Social security social workers invite potential eligible participants	48	Lone parents’ encounters with welfare to work process, in particular the most in need feeling under greatest scrutiny
McPhee &Bronstein 2003 [36]	USA	1999	Face to face interviews	All participants of (un-named) welfare to work programme	39	“Effect of welfare reforms on lone parents’ perceived ability to care for themselves and their family”
Oliker 1995 [30]	USA	1987 - 1992	Face to face interviews, observation	Participants of job search, job training programmes	30	How welfare to work lone parents make decisions about work in relation to their domestic obligations
Peacey 2009 [42]	UK	2009	Telephone interviews	Callers to helpline/participants of employability programme/internet site	34	Experience of lone parents as they move from non-conditional welfare benefits to welfare to work
Pollack and Caragata 2010 [43]	Canada	2005 - 2009	Face to face interviews	Adverts in social services offices, snowballing, referrals from welfare workers	42	“how lone mothers construct their own subjectivity” in relation to workfare
Selekman and Ybarra 2011 [35]	USA	? 2006	Face to face interviews	Random selection of participants from larger study who had increased income	15	The facilitators for welfare to work lone parents who gain paid employment

Contextual information describing the respondents’ experiences of being a lone parent and dependent on welfare benefits was provided by all of the included studies. Several studies noted that lone parents were at higher risk of role strain than two parent families [27–30], as they had less support with their domestic role, parenting duties and coping with the effects of poverty [27–31]. Combinations of circumstances, including health problems, care of extended family members, dangerous neighbourhoods, violence, frequent enforced residential moves, homelessness and domestic violence meant many lone parents struggled to cope with domestic obligations, and made trying to find and maintain work extremely difficult [29, 30, 32, 33]. Some North American studies noted that few respondents had formal qualifications [28–30, 32]. There was limited information on the age of participants across the studies. In general, the age of participants tended to range from early twenties to over 50 years. While four of the studies did not give information on the age of participants, there was no overall emphasis in the other 12 studies on young lone mothers. As Oliker observed, teenage lone mothers are usually guided towards education programmes [30].

We identified five broad themes relating to lone parents’ experiences of participating in WtW: domestic role; the WtW system; employment; economic circumstances; and health and wellbeing. The themes we identified were overlapping and at times mutually reinforcing. Insights relating to each of these key themes are presented below.

### Domestic role

Respondents’ domestic role entailed having sole responsibility for caring and providing for their children, managing their household, and organising childcare during WtW and employment activities. When WtW requirements conflicted with sole responsibility for parenting, such as lack of childcare during WtW activities, caring obligations usually took priority [27, 29, 30, 34–37]. This need to prioritise care of children could impact on participants’ ability to maintain work [28, 35], resulting in absences and financial sanctions or loss of wages [38, 39]. Within the broad theme of domestic role, there were issues of ‘parenting’, i.e. care and safety of children, which was distinct from ‘childcare’, i.e. the supervision of children by others when the parent was involved in WtW activities. These sub-themes, along with social support, are described in more detail below.

#### Parenting

There were mixed reports within studies on how WtW impacted on the participant’s role as a parent. Participation in WtW made some respondents feel they were a good role model for their children and facilitated more positive parenting [27, 33]. However, several studies noted that gaining employment could lead to conflict; while the parent could gain money and self-worth, less time was available to spend with children [27, 30, 33, 37, 39]. “*I may have more money but I don’t have more time and time is important because you can be skint and be a wonderful mother..*”([37], page 62). Exhaustion could lead to harsher parenting [27] and inability to supervise children “*There were times I came home from work and fallen asleep when she’s in a tubful of water*.” ([30], page 183).

Parents also had concerns about the safety of their children due to the requirements of WtW or subsequent employment conflicting with available childcare [30, 37, 40]. This sometimes led to older children looking after younger siblings [28, 30]. One US study reported that a participant had to leave her five year old child supervising her three year old for an hour every morning [30]. Lack of supervision for younger teenagers was a concern [29, 30], particularly when the only affordable housing was in neighbourhoods where it was unsafe for children to play outside or travel to school, and they risked coming into contact with gangs [30, 32].

#### Childcare

Difficulties finding formal or informal childcare that was affordable and safe exacerbated the challenges involved in complying with WtW requirements or subsequent employment [30, 38]. Participants experienced problems finding childcare that was: reliable and regular [27, 30, 37]; affordable [28, 30, 33, 37]; local, and flexible in order to accommodate short notice changes to hours, extra shifts, or school holidays [37]. Some respondents required specialised childcare for children with developmental or behavioural conditions [37, 38]. Lack of suitable childcare was a barrier to gaining employment [27, 30, 35, 37, 38].

#### Social support

Strong social support from family or friends, often in the form of informal childcare, was important in aiding participants to move successfully into paid employment [27, 30, 33, 35, 37]. Informal childcare was essential for some respondents, and was the only way to cope with combining unpredictable demands such as a child’s illness, with WtW or employment [30]. However, the level of social support available to respondents varied between individuals and over time [30, 35–37], with some participants having no access to social support [28, 30, 33, 39]. Even when available, informal social support could be unreliable, as the provider’s circumstances were often as unpredictable as those of the respondent [30, 33, 35].

### Welfare to work system

Several studies noted that WtW staff did not recognise the implications of being a lone parent [31, 36, 39, 41]. For example, a lone parent without child care provision was not allowed to bring her children to appointments [29, 39]. Participants often felt staff treated them with a lack of respect [29, 32, 33, 36, 37, 42, 43]. Welfare staff not fully understanding the implications of receiving various benefits caused problems as many respondents received intricate, interconnected benefits relating to their lone parent status [39]. This was exacerbated by lack of staff continuity which required respondents to explain their circumstances afresh at every appointment [37, 41, 42], short appointment slots [33], and difficulties contacting welfare staff outside regular appointments [29, 36].

There was often an emphasis on quick placement into poor quality employment [29, 30, 32, 34, 39]. One study noted a lack of appreciation of participants’ relevant skills (e.g. knowledge of children, caring roles) [40]. Training programmes that helped respondents gain basic level education [28], or computer skills [39, 42] were reported to increase respondents’ confidence. Some programmes addressed the broader problems many lone parents experienced by including methods of coping with stress [39], while others offered routes to assistance for domestic abuse [28]. However, frequently training did not lead to recognised higher qualifications and was too basic to be useful [39, 42]. Two studies reported that rather than encouraging participants to take control of their circumstances, the emphasis was on compliance with WtW requirements [39, 43]. Some respondents had little control over which WtW activities they attended [39, 41, 42].

### Employment

For some respondents, employment led to increased income [27, 37] and confidence [27, 33, 37]. Some study participants expressed ambitions for the future and a desire to work [31, 32, 36, 39, 40], *“I think I could be a social worker, a nurse, a dental assistant. I think I could do anything that involves helping people and making sure that people are happy*.”([39], page 114). However, the employment opportunities available to respondents were typically: at or near the minimum wage [30, 37]; physically demanding [30]; lacking autonomy [37]; and had limited potential for career development [33]. Many jobs involved working atypical hours outside those of regular formal childcare, inconsistent shift patterns, and long hours [27, 30, 35, 37]. Jobs were often short term [35], resulting in frequent repetition of WtW procedures including benefit applications, job searching and the upheaval of reorganising domestic arrangements to accommodate a new job [30].

Support offered by employers or co-workers could be as important as the level of pay [35], and an important factor in the sustainability of employment [37, 44]. Such support included understanding respondents’ circumstances and offering some flexibility for family related events [35, 37]. However, one study found some participants hid their lone parent status to avoid employer prejudice that lone parents were unreliable employees [31].

### Economic circumstances

Some studies reported that low income from welfare benefits caused financial insecurity for some recipients [29, 37, 39]. Routine discretionary decisions by case managers and benefit payment errors could result in sudden and unpredictable changes to essential income sources [35, 39, 41]. Low income from WtW benefits or poorly paid employment led to arrears in utility bills [42], rent payment [33, 37, 42], eviction [30], and restrictions on the family food budget [29, 31, 37, 39].

Several studies reported that even where respondents achieved full-time employment they experienced financial insecurity [27, 30, 33, 34], often relying on associated welfare benefits to meet employment incurred costs (e.g. childcare) [35]. For some, the cost of formal childcare was too high for a minimum-wage job to be economical [28, 33, 37].

Successfully achieving part-time employment that paid enough for participants to feel ‘better off’ was positive [27]. Associated in-work benefits (such as Working Tax Credit in the UK) were helpful to participants in maintaining employment [33, 37]. However, small increases in earnings could cross eligibility thresholds for other essential benefits (e.g. housing), leading to reductions in total income [30, 35, 37]. Many participants lacked any financial safety net and so were vulnerable to negative economic impacts if they lost employment or were removed from WtW [30]. The authors of two studies raised concern that inadequate income from WtW could force some respondents to turn to criminal acts to support their families [36], prevent women from leaving abusive relationships, or force participants into unsuitable relationships to obtain accommodation [29].

### Health and wellbeing

A high proportion of respondents or their children suffered from ill health which restricted their ability to take part in WtW or employment [27–29, 32, 37, 38, 41, 44]. Mental illness, depression [33, 44]; and children’s behaviour problems [30, 44] were barriers to successful participation in WtW and subsequent employment.

For some, involvement in WtW and employment exacerbated ill health [27–30, 33, 34, 37, 39, 41, 44]. While studies mentioned both physical and mental health, few studies elaborated on the effects of WtW on physical health.

Many respondents reported that participation in WtW increased stress [29, 33, 37, 39, 41]. The combined pressures of domestic obligations, involvement in WtW, employment requirements and financial insecurity were linked to poor mental health (stress, anxiety, panic attacks) [27, 33, 39, 41, 44], depression [28, 29, 34, 37, 39, 44], and fatigue [27, 30]. “*My health before work-based welfare was all right, but now…my health is not on the good side. I do be getting depressed and I am going to see a therapist for it.”* ([34], page 366).

Participation in WtW could contribute to low self-esteem and low self efficacy, the attributes respondents often required to improve their chances of gaining employment and independently supporting themselves and their families [43]. For many, WtW was experienced as stigmatising [31, 33, 37, 39, 40, 43, 44], and questions could be perceived as humiliating and intrusive [36, 39, 41, 43, 45].

There were a small number of reports of beneficial effects. WtW increased some participants’ self-worth [27, 28, 33, 37], and for some led to increased confidence in their ability to gain employment [28, 37], particularly, in one study, for those who had previous employment experience [37].

### Overarching issues of conflict and control

Across each of the descriptive themes identified, analytical themes of conflict and control emerged from participants’ reported experiences of participating in WtW and attempting to gain and maintain employment in combination with their parenting and domestic obligations.

Within the descriptive theme of domestic role, there was conflict between participants’ obligations to provide care for their children and requirements to participate in WtW activities, away from their children. Control over decisions regarding care of children was removed from respondents and dictated by WtW programmes [37, 40, 42].

Within WtW systems there was often conflict between the type of training available to respondents and what respondents required or aspired to [32, 39, 41, 42]. Respondents frequently lacked control over the type of employment applied for, with the expectation that they apply for any employment, regardless of suitability [30, 34, 37].

The nature of employment generally available conflicted with the flexibility required when raising children alone. The jobs most likely to be available to respondents (who often had few educational or vocational qualifications) offered little control over shift times and days worked, and little autonomy within the job role [27, 33–35, 37].

Problems arising from low income were frequently exacerbated by fluctuations beyond their control resulting from WtW processes. Inadequacy and fluctuation in income conflicted with participants’ need to provide adequately for their children [33, 34, 37].

The poor health and wellbeing of many respondents and their children conflicted with the requirements of WtW [27, 29, 32, 34, 37, 38, 41, 44]. This was compounded when involvement in WtW impacted on respondents’ health [27–30, 33, 34, 37, 39, 41, 44]. Trying to cope with combining both welfare activities and domestic duties could result in health issues, such as stress, fatigue and depression.

Some participants tried to overcome these conflicts and establish as much control as they were able to over their circumstances. Their priority was care of their children and they tried to fit WtW and employment commitments around their children’s needs, for example by trying to arrange WtW appointments within school hours, and seeking work suited to school hours and within easy travel of home, school, and childcare [27, 33, 37].

## Discussion

This systematic review of qualitative data provides insight into how lone parents’ involvement in mandatory WtW impacts on health and wellbeing. The potential health impacts of WtW, an upstream determinant of health, on a population vulnerable to health inequality are of international significance with the implementation of WtW policies in many high income countries. This evidence synthesis included studies from five high income countries, covering a variety of welfare regimes. We analysed data on the experiences of lone parents to uncover explanations of how participating in compulsory WtW may improve or worsen health and wellbeing. The directly conflicting demands of WtW activity and caring for children, and the loss of control over decisions regarding employment, childcare, and training, were reported to lead to stress, fatigue and poor mental health. While the majority of findings were about negative impacts of WtW, some respondents found participation in WtW a positive experience, benefiting from training and experiencing increased self-esteem.

### Strengths and limitations

The review followed a protocol and rigorous review methods, with a PRISMA checklist [24] used to guide reporting (see Additional file 3). As with all reviews, publication bias may exist; studies reporting equivocal findings may not have been published. This review included studies from five high income countries, and thus may have a relevance to other higher income countries with similar welfare programmes. However, context, such as the particular circumstances of the lone parents and the training and support provided by WtW programmes, is important to qualitative studies and should be taken into consideration when interpreting the conclusions. Several of the included studies did not detail methods of recruiting respondents, therefore we cannot rule out the possibility of selection bias through recruitment of either more disadvantaged or more successful participants. However, within the review there were diverse experiences of WtW, including participants who had succeeded in gaining employment and participants who had struggled in WtW. These diverse experiences strengthened the synthesis and interpretation of conflict and control in relation to WtW for lone parents.

### Work-family conflict for lone parents in WtW

Previous research has reported that a lack of work-life balance is associated with poor health [46], and Greenhaus and Beutells’ work-family conflict theory may help to frame the findings of our review [47]. Work-family conflict theory proposes three mechanisms through which an individual’s employment role can impact on their family role: time devoted to work; strain from participation in work; and particular behaviours required by work [47]. It has been suggested that lone mothers participating in WtW experience work-family conflict in similar ways to working mothers. This can occur when the requirements of WtW: conflict with care of children due to long or atypical hours; cause fatigue, stress, or overwhelm the participant; or impede family duties [48]. In this review, there was evidence of each of these work-family conflict mechanisms, and these were found to impact on health and wellbeing by contributing to stress, fatigue and poor mental health.

### WtW and control

Many aspects of WtW reduced participants’ ability to exercise control, particularly relating to care of children, training and employment. Lack of educational qualifications and employment experience, in addition to domestic obligations, meant participants often had little control over the type of employment available to them. This meant that many lone parents in these studies could only access precarious employment, now proposed as a social determinant of health [49]. Lack of control in these areas may link WtW participation with poor health and wellbeing. Constraints on welfare claimants’ levels of control have been found for lone parents [50] and in other welfare populations [51]. When experiencing employment insecurity, the ability of individuals to make positive changes which could improve their health can be affected by their perceived control as well as structural factors [52]. Lack of perceived control of circumstances has been connected to poor health through psychological and biological pathways [53–55]. Lack of control may trigger chronic stress, leading to negative emotions and depression [53–55]. These conditions may also lead to negative biological impacts on the immune and cardiovascular systems [56].

This review found that many participants were unable to gain control of their circumstances and reported poor wellbeing, particularly stress. For some respondents, taking control of their lives involved removing themselves, temporarily or long term, from WtW, as they could not maintain sufficient care for their child [30, 36]. This is consistent with evidence of increasing ‘disconnection’ from work or welfare in the United States. In 2011/12, over five million children in the US lived in disconnected families [57], that is, with parents who are neither in work nor in receipt of welfare, and have no known source of income. There is some evidence that this is beginning to occur in the UK [58].

For lone parents who benefitted from WtW, higher control was facilitated through enhanced skills or qualifications, increased confidence in their employability, accessing employment that was compatible with caring responsibilities, and earnings sufficient to improve their standard of living. It may be that lone parents with positive experiences of WtW have less conflict to manage and greater control of their circumstances. Social support allowed some participants to manage conflicts between WtW and bringing up children alone. Positive social support might contribute towards a reduced level of conflict. Observational studies have found evidence that social support can assist in managing work-family conflict [59], and that supportive workplace practices increase perceived control and reduce conflict, resulting in lower rates of depression, blood cholesterol and other complaints [60]. However, social support can have negative impacts, expectations of reciprocal support (see review [61]), and as found in this review, many lone parents do not have a consistent support network.

## Conclusion

This synthesis of the experiences of lone parents in mandatory WtW suggests that WtW participation may do little to improve lone parents’ health and wellbeing or economic circumstances, often only leading to low paid, precarious employment. Conflict and control appear to be mechanisms that link lone parents’ participation in WtW with health. The demands of parenting alone and employment are frequently in direct conflict, and lone parents are often denied control over major life decisions and everyday routines by WtW obligations.

While WtW may have potential to contribute towards improving health and wellbeing for lone parents, contextual mediating factors may act to counter this potential. In particular, unavailability of suitable employment, welfare assistance, childcare, and social support, may lead to WtW being counterproductive with respect to health and wellbeing. As employment requirements for lone parents in receipt of welfare are implemented internationally, increased awareness of the adverse impacts for many, and the potential for negative impacts on health and wellbeing due to the conflicts inherent in combining employment with raising children alone, may help to develop more effective interventions. WtW programmes which do not provide adequate training, emphasise placement in any available job, and do not recognise individual circumstances such as health problems are unlikely to lead to improved economic security and may be counterproductive for the health of lone parents. Therefore, while acknowledging the limitations discussed above, our recommendation based on the findings of this review are for further research on the health and wellbeing implications for lone parents of participating in mandatory WtW. In particular, there should be further investigation of how this vulnerable population can gain greater control of their circumstances, and how conflicts between lone parenthood and mandatory WtW can be resolved.

## Additional files

Additional file 1:
**Bibliographic databases searched, search terms and example search.** (DOCX 16 kb) Additional file 2:
**Quality assessment.** (DOCX 36 kb) Additional file 3:
**PRISMA checklist.** (DOC 63 kb)
