# Supplementary material for: Lone parents, health, wellbeing and welfare to work: a systematic review of qualitative studies
Source: BMC Public Health. 2016 Feb 25;16:188. doi: 10.1186/s12889-016-2880-9 (PMC4766630; doi:10.1186/s12889-016-2880-9)
Supplement: Additional file 1: — Bibliographic databases searched, search terms and example search. (DOCX 16 kb) [file 12889_2016_2880_MOESM1_ESM.docx]

# Lone parents, welfare to work interventions and health: synthesis of qualitative studies

**Additional file 1: Bibliographic databases searched and example search**

**Bibliographic databases searched**

- MEDLINE
- Embase
- ERIC
- Cinahl
- EconLit
- IBSS
- PsycINFO
- SocINDEX
- Social Services Abstracts
- Sociological Abstracts
- ASSIA
- Web of Science
- Cochrane Database of Systematic Reviews
- ProceedingsFirst
- PapersFirst
- EThOS‎
- Theses Canada
- WorldCatDissertations
- Proquest Dissertations & Theses
- Australasian Digital Theses
- OpenSIGLE

**Example search**

Ovid MEDLINE(R) 1950 to July Week 2 2009

16.7.9

1. (Lone mother* or Lone parent* or One parent* or Single mother* or Single parent* or Single-parent* or sole mother* or Sole parent* or Unwed Mother*).af.

limit 1 to humans

3675 hits

2. ((Active labour market polic* or Active labor market polic* or Active labour market programme* or Active labor market programme* or ADFC or Agenda 2010 or Aid to Families with Dependent Children or Allocation Parent Isole or ALMP) and America Works) or API or (Canada Health and Social Transfer) or CHST or Community Wage or Domestic Purposes Benefit or Employment Tax Deduction or ETD or Family Transition Programme or FTP or Hilfe zum Arbeit or Hilfe zum Lebensunterhalt or Individual Re-integration Agreement or IRO or Jobbskatteavdraget) and Labour market activation and Labor market activation) or MFIP or Minnesota Family Investment Program or National childcare allowance or National Evaluation of Welfare-to work Strategies or NDLP or New Deal for Lone Parents or New Hope Project or Newstart allowance or NEWWS or Ontario Works or (Personal Responsibility and Work Opportunity Reconciliation Act) or PRWORA or Revenu Minimum d Insertion or RMI or Self- Sufficiency Project or Social assistance or SSP or TANF or Temporary Assistance for Needy Families or Work for your dole or workfare or Working For Families or Back-to-work or Employment or Employment Program* or Family Program* or Financial support or Government program* or Income support or Job or Jobless* or Poverty or public welfare reform or Retrain* or Re-train* or Social assistance or Social security or Social welfare* or Training or Unemployment or Vocation* or Welfare or welfare polic* or Welfare reform* or Welfare to work or Work or Workless*).af.

limit 3 to humans

510783

1 AND 2 = 10
