# Supplementary material for: Lone parents, health, wellbeing and welfare to work: a systematic review of qualitative studies
Source: BMC Public Health. 2016 Feb 25;16:188. doi: 10.1186/s12889-016-2880-9 (PMC4766630; doi:10.1186/s12889-016-2880-9)
Supplement: Additional file 2: — Quality assessment. (DOCX 36 kb) [file 12889_2016_2880_MOESM2_ESM.docx]

**Lone parents, welfare to work interventions and health: synthesis of qualitative studies.**

**Additional file 2**

**Quality assessment**

| **Author** | **Research questions/aim specified?** | **Research questions suited to qualitative enquiry?** | **Sampling clearly described? (does it tell how chose the participants?)** | **Data collection clearly described?** | **Analysis clearly described?** | **Sampling appropriate to research question?** | **Data collection appropriate to research question?** | **Analysis appropriate to research question?** | **Does the paper make a useful contribution to the review question? (assessed in light of answers to previous questions)** |
| --- | --- | --- | --- | --- | --- | --- | --- | --- | --- |
| Baker 2002;  Baker & Tippin 2002, 2004 | yes | yes | Yes (in B&T 2002) | Yes (in 2004) | no | yes | yes | n/a | yes |
| Breitkreuz et al. 2010 | yes | yes | yes | yes | yes | yes | yes | yes | yes |
| Critelli et al. 2010 | yes | yes | yes | yes | yes | yes | yes | yes | yes |
| Good Gingrich 2010 | yes | yes | no | yes | yes | n/a | yes | yes | yes |
| Grahame & Marston 2012 | yes | yes | yes | no | yes | yes | n/a | yes | yes |
| Haux et al. 2012 | yes | yes | yes | yes | yes | yes | yes | yes | yes |
| Hildebrandt 2002; Hildebrandt & Kelber 2005 | yes | yes | yes | yes | yes | yes | yes | yes | yes |
| Hildebrandt 2006 | yes | yes | yes | yes | yes | yes | yes | yes | yes |
| Hildebrandt & Ford 2009 | yes | yes | yes | yes | yes | yes | yes | yes | yes |
| Lane et al. 2011 | yes | yes | yes | yes | yes | yes | yes | yes | yes |
| McArthur et al. 3013 | yes | yes | yes | yes | yes | yes | yes | yes | yes |
| McPhee &Bronstein 2003 | yes | yes | yes | yes | yes | yes | yes | yes | partially |
| Oliker 1995 | yes | yes | yes | yes | no | yes | yes | n/a | yes |
| Peacey 2009 | yes | yes | yes | yes | yes | yes | yes | yes | yes |
| Pollack & Caragata 2010 | yes | yes | yes | yes | yes | yes | yes | yes | partially |
| Selekman and Ybarra 2011 | yes | yes | yes | yes | yes | yes | yes | yes | yes |

Questions based on Dixon Woods (2004) (n/a not applicable)
